# Cell-autonomous effect of cardiomyocyte branched-chain amino acid catabolism in heart failure in mice

**DOI:** 10.1038/s41401-023-01076-9

**Published:** 2023-03-29

**Authors:** Jia-yu Yu, Nancy Cao, Christoph D. Rau, Ro-Po Lee, Jieping Yang, Rachel J. Roth Flach, Lauren Petersen, Cansheng Zhu, Yea-Lyn Pak, Russell A. Miller, Yunxia Liu, Yibin Wang, Zhaoping Li, Haipeng Sun, Chen Gao

**Affiliations:** 1 Key Laboratory of Cell Differentiation and Apoptosis of Ministry of Education, Department of Pathophysiology, Shanghai Jiao Tong University of Medicine, Shanghai, 200025 China; 2 School of Medicine and Public Health, University of Wisconsin, Madison, WI USA; 3 Department of Genetics, School of Medicine, University of North Carolina, Chapel Hill, NC USA; 4 Department of Medicine, David Geffen School of Medicine, University of California, Los Angeles, CA USA; 5 Pfizer Worldwide Research, Development and Medical, Cambridge, MA USA; 6 Health Science Center, University of Utah, Salt Lake City, UT USA; 7 Department of Pharmacology and Systems Physiology, University of Cincinnati, Cincinnati, OH USA; 8 Department of Anesthesiology, David Geffen School of Medicine, University of California, Los Angeles, CA USA; 9 La Ronde Pharmaceuticals, Cambridge, MA USA; 10 Signature Research Program in Cardiovascular and Metabolic Diseases, DukeNUS School of Medicine and National Heart Center of Singapore, Singapore, Singapore

**Keywords:** branched-chain amino acid, metabolism, heart failure

## Abstract

Parallel to major changes in fatty acid and glucose metabolism, defect in branched-chain amino acid (BCAA) catabolism has also been recognized as a metabolic hallmark and potential therapeutic target for heart failure. However, BCAA catabolic enzymes are ubiquitously expressed in all cell types and a systemic BCAA catabolic defect is also manifested in metabolic disorder associated with obesity and diabetes. Therefore, it remains to be determined the cell-autonomous impact of BCAA catabolic defect in cardiomyocytes in intact hearts independent from its potential global effects. In this study, we developed two mouse models. One is cardiomyocyte and temporal-specific inactivation of the E1α subunit (*BCKDHA-cKO*) of the branched-chain α-ketoacid dehydrogenase (BCKDH) complex, which blocks BCAA catabolism. Another model is cardiomyocyte specific inactivation of the BCKDH kinase (*BCKDK-cKO*), which promotes BCAA catabolism by constitutively activating BCKDH activity in adult cardiomyocytes. Functional and molecular characterizations showed E1α inactivation in cardiomyocytes was sufficient to induce loss of cardiac function, systolic chamber dilation and pathological transcriptome reprogramming. On the other hand, inactivation of BCKDK in intact heart does not have an impact on baseline cardiac function or cardiac dysfunction under pressure overload. Our results for the first time established the cardiomyocyte cell autonomous role of BCAA catabolism in cardiac physiology. These mouse lines will serve as valuable model systems to investigate the underlying mechanisms of BCAA catabolic defect induced heart failure and to provide potential insights for BCAA targeted therapy.

## Introduction

Branched-chain amino acids (BCAA), including valine, leucine and isoleucine, are basic building blocks for all life-forms [1]. In animal cells, BCAA are essential nutrient since they cannot be generated from *de novo* biosynthesis, and the homeostasis of BCAA is regulated by food uptake and cell degradation via a highly conserved catabolic pathway. BCAA is first catabolized into branched-chain α-ketoacids (BCKAs) by branched-chain amino-transferase (BCAT) in a reversible manner; while the second and the rate limiting step is carried out by branched-chain α-keto dehydrogenase complex (BCKDH) consisting of E1α, E1β, E2 and E3 catalytic subunits encoded by BCKDHA, BCKDHB, BCKDE2, BCKDE3 genes, respectively. Similar to Pyruvate Dehydrogenase (PDH), BCKDH activity is potently regulated by specific phosphorylation on E1α subunit by a BCKD kinase (BCKDK) [2] and dephosphorylation by a mitochondria phosphatase, PP2Cm. While rare genetic defects in BCKDH are the genetic basis of Maple Syrup Urine Disease, a neurological disease with fatal outcome, recent findings also link BCAA catabolic disorder with a variety of common human diseases, including cancer and diabetes [3–5]. Therefore, global and cell specific BCAA homeostasis is critical for normal development and physiology across different tissues.

Recent metabolomics and transcriptomic analysis have revealed that the deficiency in BCAA catabolism is an important metabolic feature in failing hearts from animal models to humans [4, 6–9]. While genetic inactivation of PP2Cm leads to global impairment of BCKDH activity and promotes heart failure in ageing or in response to pathological stresses [4, 10], restoring BCKD activity via administration of a highly specific BCKDK inhibitor (BT2) is shown to ameliorate cardiac dysfunction and pathogenic progression of heart failure [11, 12]. Thus, BCAA catabolic defect not only directly contributes to the onset of heart failure, but also can be targeted for potential therapy.

Beyond their role as essential protein building blocks and very limited contribution to overall bioenergetics, BCAA has also been implicated to have various metabolic and physiologic impact as nutrient signaling molecules by targeting different cellular pathways [13, 14]. A classic and well-documented cellular signaling for BCAA mediated regulation of cell growth and autophagy is mammalian Target of Rapamycin (mTOR) pathway. Indeed, mTOR activity has been well implicated in cardiac hypertrophy and pathological remodeling in heart [15, 16]. In addition, BCAA catabolism is reported to affect lipid and glucose metabolism, insulin production and cell differentiation in skeletal muscle, liver, pancreas and adipose tissue [4, 17–21]. Importantly, recent studies from clinical cohort and animal models have revealed a profound impact of systemic BCAA catabolic defect on the pathogenesis of insulin resistance and diabetes, particularly associated with obesity [22, 23]. Pharmacological treatment targeted to enhance BCAA catabolic flux has been reported to significantly improve systemic insulin signaling in models of type-2 diabetes with obesity [24]. Loss of insulin signaling is another important molecular and cellular signature in failing heart [25, 26]. However, it remains to be determined if BCAA catabolic defect has direct deleterious impact on cardiac function in a cardiomyocytes cell-autonomous manner or affects heart through systemic impairment of global metabolic activities. Furthermore, a recent report by Murashige et al. highlights the potential role of extra-cardiac BCAA catabolism in cardioprotection against cardiac injury and dysfunction [11], raising additional questions about the cellular mechanism of the cardioprotective effect conferred by enhancing BCAA catabolic activity.

To address this important question and overcome the limitation in previous studies using mice with global PP2Cm knockout or systemic administration of BCKDK inhibitor [4], we have developed two mouse models with inducible and cardiomyocyte-specific manipulation of BCAA catabolic pathway. One is inactivation of *bckdha* gene which encodes the E1α subunit of the BCKDH holoenzyme complex, rendering complete blockage of BCAA catabolic flux. The other is depletion of *bckdk* gene which encodes the kinase phosphorylating and inactivating the E1α subunit of the BCKDH complex, thus conferring maximum catabolic flux of BCAA in cardiomyocytes. Functional and molecular analysis showed that cardiomyocyte specific impairment of BCKDH activity led to rapid and profound contractile defects in intact mouse heart associated with significant reprogramming in cardiac transcriptome. Surprisingly, constitutive activation of BCAA catabolism in heart through depleting *bckdk* gene had no discernible impact on cardiac function either at baseline or under pressure overload stress. These results support a significant myocyte-specific cell-autonomous role of BCAA catabolic defect in the pathogenesis of heart failure, but also raise some interesting questions for the cellular mechanisms involved in the previously well characterized cardioprotective effects from pharmacological activation of BCAA catabolism. In summary, these two mouse lines characterized here may serve as important model systems to investigate myocyte specific cell-autonomous effect of BCAA catabolic activities in heart failure.

## Materials and methods

### Animals and tamoxifen treatment

The mBckdha and mBckdk floxed allele were generated by Cyagen (https://www.cyagen.com/us/en/) through traditional ES clones selection followed by blastocyst microinjection. The *bckdha*^*flox/flox*^ mouse line contains two LoxP sites flanking the exon 4 of the *mbckdha* gene on Chromosome 7. The *bckdha*^*flox/flox/cre*^ mice (BCKDHA-cKO) were generated by crossing the mice carrying *bckdha*^*flox/flox*^ allele with mice carrying αMHC-Mer-Cre-Mer transgene [27] where the *mbckdha* gene can be inactivated upon tamoxifen treatment (Supplementary Fig. 1). The *bckdk*^*flox/flox*^ mouse line contains two LoxP sites flanking the exons 2–8 of the *mbckdk* gene on Chromosome 7. The *bckdk*^*flox/flox/cre*^ mice (BCKDK-cKO) were generated by crossing the *bckdk*^*flox/flox*^ mice with αMHC-Mer-Cre-Mer mice [27] where the Bckdk can be inactivated in cardiomyocytes upon tamoxifen treatment (Supplementary Fig. 3). The genotype was confirmed by both Southern Blot and genomic DNA PCR. The genetic background of all strains is C57BL/6 J. Baseline (Day 0) echocardiography was recorded in mice at 8 weeks of age prior to five daily intraperitoneal injections of tamoxifen at 40 mg/kg bodyweight. All animals in this study were handled in accordance with the Guide for the Care and Use of Laboratory Animals published by the US National Institutes of Health.

### Echocardiogram recording

Echocardiogram analysis for mice was performed as previously described [28]. Briefly, mice were anesthetized and maintained with 2% isoflurane in 95% oxygen. Ultrasound images were recorded using Vevo 2100 (Visual Sonics) equipped with a 30 mHZ scan head. The short axis views at mid-section marked by papillary muscle were used to generate M mode images and to calculate cardiac parameters.

### Protein and mRNA analysis

Immunoblots were performed as described [28] using the following primary antibodies: anti-BCKDHA (abcam, ab138460), anti-BCKDK (santa cruz, sc-374425), anti-DBT (ThermoFisher, PA5-29727), anti-BCKDHA (phosphor S293) (Abcam ab200577), anti-GAPDH (Cell Signaling #2118). Total RNA was prepared using Trizol Reagent (Invitrogen). Following reverse-transcription, real-time qPCR reactions were performed in triplicate wells and were quantified by CFX96 Real-Time PCR system (BioRad). Signals for β-actin gene (ACTB) and GAPDH were used for normalization. The sequences for the RT-PCR primers were as follows (5'-3'):

mANF RT-F: AGGCAGTCGATTCTGCTTGA;

mANF RT-R: CGTGATAGATGAAGGCAGGAAG;

mBCKDHA RT-F: ACATGACCAACTATGGCGAGG;

mBCKDHA RT-R: CCGGTACATGAGCACACCTG;

mBCKDK RT-F: GCTATACATCCGGGCCTTCC;

mBCKDK RT-R: GGATGTGTTTCCGGCTCTCA;

mACTB RT-F: TGGCACCACACCTTCTACAA;

mACTB RT-R: GTCTCCGGAGTCCATCACAA;

mBNP RT-F: GAAGGTGCTGTCCCAGATGATT;

mBNP RT-R: GCTCTGGAGACTGGCTAGGACTT;

mGAPDH RT-F: ACCCAGAAGACTGTGGATGG;

mGAPDH RT-R:CACATTGGGGGTAGGAACAC;

mBCKDE2 RT-F: GCTCAGGAAAAGATGGCAGAA;

mBCKDE2 RT-R: TTTGGGCTGTGGTGGAGGT;

mMyh7 RT-F: GTTTGTCAAGGCCAAGATCGTGT;

mMyh7 RT-R: AGCATGGCCATGTCCTCGAT

Genotyping primers:

*bckdha* flox F: GTCCCTCTCTCTGTGTCCCT;

*bckdha* flox R: CTCCTGGCCCTCACTGATTG;

bckdk flox F: CCTCTCCATCTTCTTAATGCTGGG;

bckdk flox R: GTCAGTAATAGGGGGATGGAGAGAT

### WGA staining

Hearts were harvested under anesthesia and perfused with PBS followed by fixation in 4% formalin at 4 ºC for 24 h and subsequently imbedded in paraffin section. The tissue sections were deparaffined and rehydrated before blocking in 10% species specific serum in 1%BSA/PBS for 1 h at room temperature. The Alexa Fluor 594 conjugated WGA (5 mg/ml, Life Technologies W11262) were diluted in PBS and incubated with the section for 1 h at room temperature and protected from the light. The samples were counterstained with DAPI (Life Technologies, D3571) and mounted with SlowFade Goat Antifade Reagent (Life Technologies S36936). The WGA sections were visualized under Nikon Fluorescent microscope and the cross-section area was quantified using Image J.

### RNA-sequencing and bioinformatics analysis

Total RNA was extracted from left ventricle tissue of 8 weeks old male (*n* = 3) and female (*n* = 3) of BCKDHA-cKO and Control mice 1 week after completion of Tamoxifen treatment. RNA-sequencing was performed, and the raw reads were collected at UCLA Clinical Microarray Core. Transcript abundances were calculated using the 0.8.2 version of the Salmon algorithm [29] by pseudoalignment to the Ensembl Mus Musculus GRCm38 build of the mouse transcriptome with correction for both sequence and GC biases in the reads. Principle Component Analysis was performed on all expressed and varying genes (average TPM > 1, Coefficient of Variation >5%) using the prcomp R function and the ggbiplot R package (R package version 0.55. http://github.com/vqv/ggbiplot). Differentially expressed genes were calculated using DESEQ2 [30] R package, and genes were selected for further study that met all of the following criteria: Average TPM >1, absolute fold change >1.5, and FDR corrected *P* value <0.05. Integrated pathway and Gene Ontology Enrichment analyses were performed with clueGO (https://pubmed.ncbi.nlm.nih.gov/19237447/).

### BCAA and BCKA measurement

The mice were starved for a total of 4 h before given a single dose of BCAA mixture (BCAA amount: 1.5 mg/g BW, isoleucine:leucine:valine = 0.8:1.5:1 by weight) via i.p injection. Mice were euthanized 30 min post BCAA mixture challenge before tissue/serum were collected. Briefly, mouse cardiac tissue was pounded to powder under liquid N_2_ and homogenized and sonicated in working internal standard solution (WISS) containing Leucine D_10_, Isoleucine D_10_, Valine D_8,_ Ketoleucine D_3_, Ketoisoleucine D_8,_ Ketovaline ^13^C_4_ D_4_ in 4:1 methanol/water at a ratio of 50 mg tissue/1 mL WISS. Samples were spun, and the supernatant was collected and transferred to a deep well injection plate followed by drying under nitrogen at 40 °C. Residue was resuspended in 50 µL water containing 0.2% acetic acid. Samples were run on a Dionex Ultimate 3000 with a Waters HSS T3 1.8 µm, 2.1 × 100 mm column. The mobile phase was A:1000:2 water/acetic acid and B: methanol. Gradient separation began at 1.8% B for 2 min then increased to 5.5% B over 0.5 min, then to 6.5% B over 2.75 min, then to 100% B over 1.75 min and held for 1 min followed by a return to 1.8% B and held for 1 min. Data were acquired on a Thermo Q-Exactive mass spectrometer within a scan range of 100-160 *m*/*z*.

### Statistical analysis

Statistical analysis to compare 2 groups was performed using the Student *t*-test. When more than 2 groups were analyzed, ANOVA will be performed. Presented values are mean with standard deviation of the mean. A value of *P* < 0.05 was considered statistically significant.

## Results

### Cardiac specific inactivation of Bckdha in adult mouse heart

**Fig. 1:**
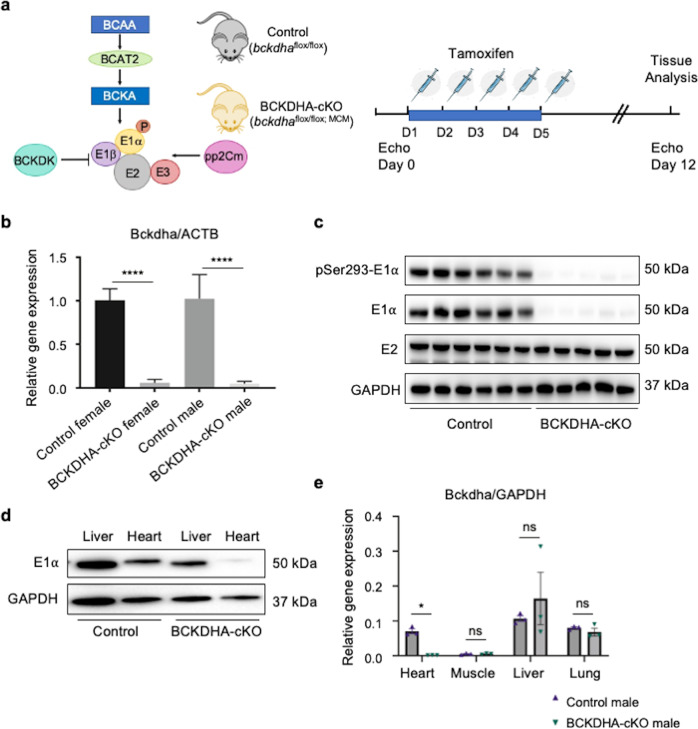
Generation of cardiac specific BCAA metabolism defective mouse model using BCKDHA-cKO mice. **a** Schematic view of BCKDH complex in BCAA catabolic pathway and the experimental design. **b** Bckdha mRNA levels in the Control and the BCKDHA-cKO mouse hearts measured by real-time RT-PCR, *n* = 7–10 each group, ****P < 0.001. **c** Representative immunoblot data of total and phosphorylated E1α protein, and E2 protein in the BCKDHA-cKO and the Control mouse hearts. **d** Representative immunoblot data of E1α expression in liver and heart tissues from the Control and the BCKDHA-cKO mice. **e** Real-tim**e** PCR analysis of BCKDHA normalized to GAPDH in different tissues as indicated. *n* = 3 each group, *P < 0.05.

A mouse strain with *bckdha* gene floxed at exon 4 (*bckdha*^*flox/flox*^) was generated in C57BL/6 J background. By crossing *bckdha*^*flox/flox*^ mice with αMHC-Mer-Cre-Mer (*MCM*) transgenics [27], we generated *bckdha*^*flox/flox*^:*MCM* (BCKDHA-cKO) mice. Cardiomyocyte specific inactivation of *bckdha* gene was induced in 8 weeks old adult BCKDHA-cKO mice by tamoxifen administration for five consecutive days, along with the same treatment in the *bckdha*^*flox/flox*^ littermates as controls (Fig. 1a, Supplementary Fig. 1). One week after completion of tamoxifen administration (12 days post tamoxifen administration), the bckdha mRNA and E1α protein were markedly reduced in the BCKDHA-cKO hearts compared to the Controls (Fig. 1b, c), while its expression in the liver, skeletal muscle and lung tissues was not affected (Fig. 1d, e). Therefore, BCKDHA-cKO mice showed efficient and specific Bckdha inactivation upon tamoxifen treatment.

### Functional impact of cardiomyocyte specific BCAA catabolic defect

**Fig. 2:**
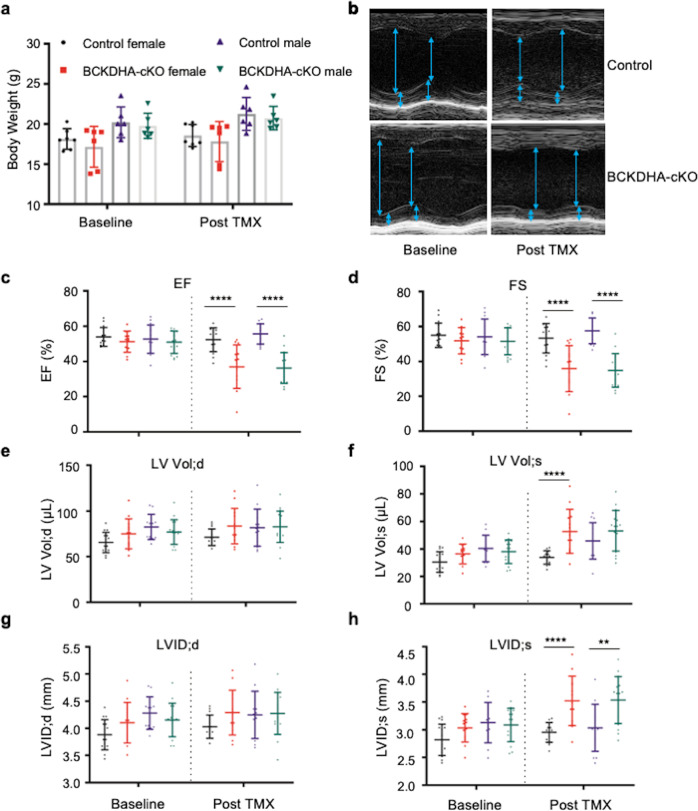
Impaired cardiac function following BCAA catabolic defect in heart. **a** Body weight of the male and the female Control and BCKDHA-cKO mice at baseline (Day 0) and one week post tamoxifen injection (Day 12). **b** Representative echo M-mode images for the Control and the BCKDHA-cKO mice at baseline and post tamoxifen mediated gene inactivation. **c**–**h** Echocardiography parameters, including (**c**) ejection fraction (EF%); (**d**) fractional shortening (FS%); (**e**) left ventricle end-diastolic volume (LV Vol;d); (**f**) left ventricle end-systolic volume (LV Vol;s); (**g**) left ventricle end-diastolic internal diameter (LVID;d) and (**h**) left ventricle end-systolic internal diameter (LVID;s) in the Control and the BCKDHA-cKO male and female animals at baseline (Day 0) and one week post-tamoxifen induced gene inactivation (Day 12). **P < 0.01; ****P < 0.001.

We followed both Control and the BCKDHA-cKO male and female mice at baseline and post tamoxifen administration and found no difference in body weights between the different genotype groups (Fig. 2a). Echocardiography before (Day 0) and one week after tamoxifen administration (Day 12) were acquired (Fig. 2b–h). Compared to the Controls, cardiomyocyte specific inactivation of *Bckdha* led to a significant decrease in cardiac contractile function (Fig. 2b) characterized by reduced ejection fraction (EF, Fig. 2c) and fractional shortening (FS%, Fig. 2d) in both male and female cohorts. While left ventricular end-diastolic volume and diameter were not affected by Bckdha inactivation (Vol:d, Fig. 2e, LVID:d, Fig. 2g), the end-systolic volume and diameter were significantly increased in the BCKDHA-cKO hearts (Vol;s, Fig. 2f, LVID:s, Fig. 2h), indicating a state of systolic dysfunction.

### Cardiac BCAA metabolism defects led to pathogenic remodeling in vivo

**Fig. 3:**
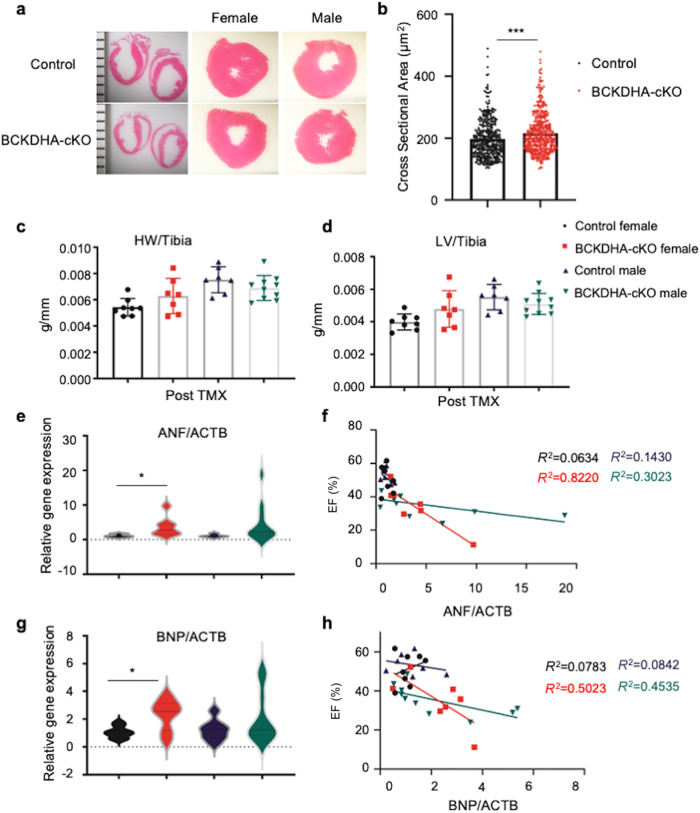
Pathological remodeling following BCAA catabolic defect in heart. **a** Representative H&E staining of the Control and the BCKDHA-cKO male and female mice cardiac section. Magnification: 10×. **b** Cardiomyocytes cross-section area in the Control and the BCKDHA-cKO hearts, ***P < 0.005. **c**, **d** Heart weight vs. tibial length (HW/TL), Left ventricle weight vs. tibial length (LV/TL) in the Control and the BCKDHA-cKO animals. **e**–**h** mRNA levels of ANF (**e**, **f**) and BNP relative to ACTB (**g**, **h**) in the Control and the BCKDHA-cKO male and female mouse hearts (**e**, **g**) as well as their quantitative correlation with cardiac function in each cohort (**f**, **h**).

To further understand the impact of impaired cardiac BCAA metabolism, we analyzed the heart morphology in the Control and the BCKDHA-cKO mouse hearts. Morphometric analyses showed no significant chamber thickening or gross changes in chamber morphologies (Fig. 3a), however we indeed observed a moderate but statistically significant increase in cardiomyocytes cross section area based on WGA staining (Fig. 3b). In agreement with the histological observations, no significant differences were observed in heart weight or left ventricle weight between the BCKDHA-cKO mice and the controls in either sex group (Fig. 3c, d). At molecular level, inactivation of *Bckdha* in heart caused a modest but significant induction of cardiac stress marker genes, including ANF and BNP, especially in female BCKDHA-cKO hearts and the magnitudes of their induction correlated with the severity of cardiac dysfunction (Fig. 3e–h). Interestingly, we did not observe a similar change of Myh7 mRNA expression in these BCKDHA-cKO hearts for both genders (Supplementary Fig. 2). These results represent the first in vivo evidence that loss of BCAA catabolic activity in adult cardiomyocytes is sufficient to cause a rapid onset of cardiac dysfunction in the absence of gross remodeling. These results are consistent with earlier observations from reports using PP2Cm KO or BCKDK inhibition that BCAA catabolic activity is highly associated with cardiac function but with limited if any impact on cardiac hypertrophy [28].

### BCAA catabolic defect on metabolic homeostasis

**Fig. 4:**
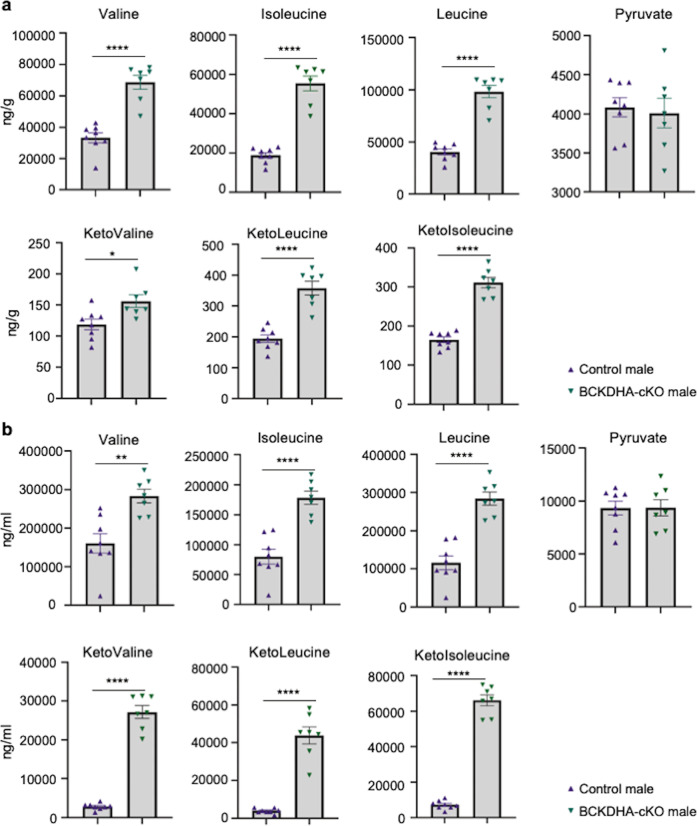
Impaired cardiac BCAA metabolism led to changes of systemic BCAA/BCKA. **a** BCAA, pyruvate and BCKA concentrations in the Control and the BCKDHA-cKO mouse cardiac tissues collected at one week post tamoxifen administration. **b** BCAA, pyruvate and BCKA concentrations in the Control and BCKDHA-cKO mouse serum samples collected at one week post tamoxifen administration. *P < 0.05, **P < 0.01, ****P < 0.001.

To understand how tissue-specific inactivation of BCKDHA could impact on BCAA oxidation process in heart, we performed direct measurement of BCAA and BCKA species using mass-spectrometry in cardiac tissues and plasma from the BCKDHA-cKO and Control mice 30 min after a single dose of BCAA challenge. As expected, inactivation of E1α in cardiomyocytes impaired BCAA clearance in heart tissue without affecting the pyruvate concentration, reflecting defective BCAA catabolic flux (Fig. 4a). Unexpectedly, similar changes in plasma levels of BCAA and BCKA were also observed in the BCKDHA-cKO mice, both BCAA and BCKA were significantly increased in the BCKDHA-cKO mice plasma without affecting pyruvate concentration (Fig. 4b). To our knowledge, this is the first evidence illustrating cardiac specific inactivation of BCKDH could impact on global BCAA metabolism at systemic level.

### Cardiac specific inactivation of Bckdk in adult mouse heart

**Fig. 5:**
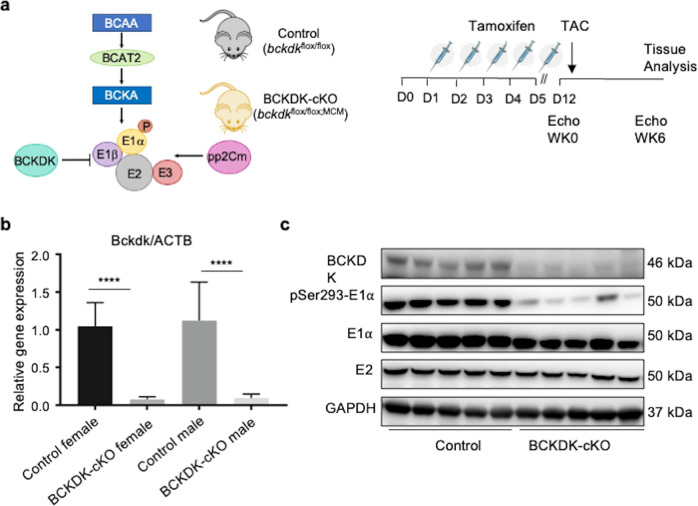
Generation of cardiac specific BCAA metabolism active mouse via BCKDK-cKO mouse line. **a** Schematic view of the BCKDK-cKO mouse line generation and experimental design. **b** Real-time PCR analysis of BCKDK mRNA expression level in the Control and the BCDK-cKO male and female mouse left ventricle tissues. *n* = 4–5 each group, ****P < 0.001. **c** Western Blot analysis of BCKDK, E1α, phosphor-E1α (S293) and E2 expression level in the Control and the BCKDK-cKO mouse left ventricle tissues, GAPDH was used as control.

Earlier results have shown pharmacological enhancement of branched-chain α-keto acid dehydrogenase activity significantly blunted cardiac dysfunction under pressure overload [11, 12]. To further illustrate whether the protective effect was due to cell-autonomous effect of BCKDH activation, we generated a cardiomyocyte specific BCKD kinase knockout mouse model (BCKDK-cKO) to achieve cardiac specific branched-chain α-keto acid dehydrogenase complex activation. As illustrated in Fig. 5a (Fig. 5a, Supplementary Fig. 3), the BCKDK flox allele contains loxP site flanking the exons 2–8 of the mouse *bckdk* gene, and the cardiac specific BCKDK inactivation was achieved by crossing the *bckdk*^flox/flox^ with αMHC-Mer-Cre-Mer mouse line. Both the Control and the BCKDK-cKO mice received 5 consecutive days of tamoxifen administration prior to a 7-day washout period before echocardiography was performed. Following echocardiogram acquisition at baseline, transaortic constriction (TAC) was performed and the mice were followed for a duration of 6 weeks post TAC using echocardiography. Based on both mRNA and protein analysis, BCKDK, but not E1α was efficiently depleted in the BCKDK-cKO mice left ventricle tissues compared to the Control (Fig. 5b, c).

### Genetic activation of branched-chain α-keto dehydrogenase complex does not prevent heart failure progression induced by pressure overload

**Fig. 6:**
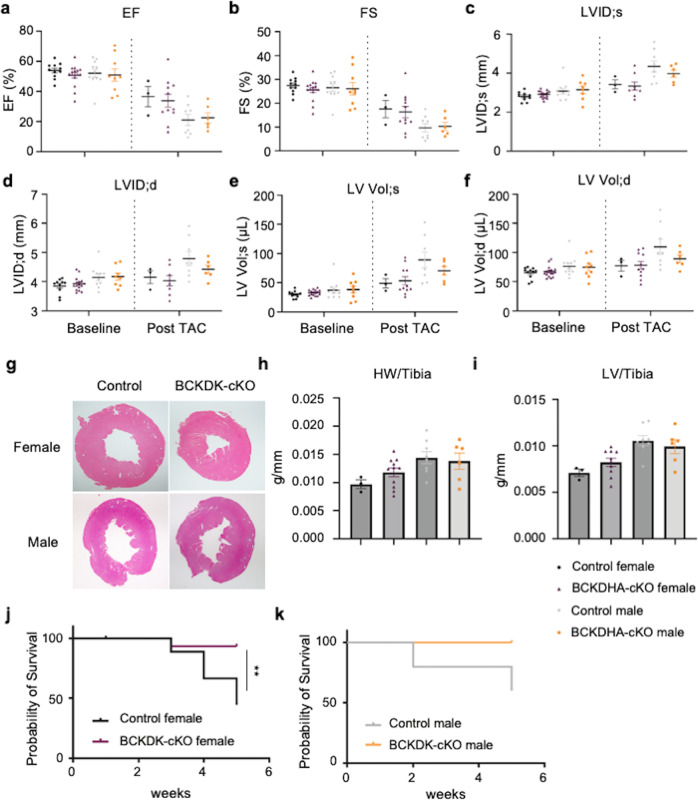
Inactivation of BCKDK in heart does not prevent heart failure post pressure overload. **a**–**f** Echocardiography parameters including Ejection Fraction (**a**), Fraction Shortening (**b**), LVID;s (**c**), LVID;d (**d**), LV Vol;s (**e**) and LV Vol; d(**f**) in the Control and the BCKDK-cKO male and female mice, at baseline and 6 weeks post pressure overload injury. **g** Representative H&E staining for the Control and the BCKDK-cKO female and male mouse hearts post pressure overload surgery. Magnification: 10×. **h** Heart weight/tibia length ratio in the Control and the BCKDK-cKO male and female mice 6 weeks post TAC. **i** Left ventricle weight/tibia length ratio in the Control and the BCKDK-cKO male and female mice 6 weeks post TAC. **j**–**k**: Survival curve for the Control and the BCKDK-cKO female (**j**) and male (**k**) mice post TAC at indicated time points. **P < 0.01.

Cardiac function in the Control and the BCKDK-cKO mice were measured at baseline and 6 weeks post pressure overload using echocardiography. At baseline, inactivation of *Bckdk* did not lead to any significant change in cardiac function based on both Ejection Fraction (Fig. 6a) and Fraction Shortening (Fig. 6b). At week 6 post pressure overload, the BCKDK-cKO female and male mice developed similar degrees of cardiac dysfunction compared to their corresponding Control cohorts as measured from ejection fraction, fractional shortening, end-diastolic and end-systolic volumes (Fig. 6a–f). At tissue morphological level, inactivation of BCKDK did not change the degree of cardiac hypertrophy in response to pressure overload in either male or female cohort based on histology (Fig. 6g), heart weight (Fig. 6h) or left ventricle weight /tibia length (Fig. 6i). However, the female BCKDK-cKO mice showed a significant better survival rate compared to the control female (Fig. 6j), the survival rate also trended better in male BCKDK-cKO mice compared to the Control males (Fig. 6k). In summary, cardiomyocyte specific inactivation of BCKDK is not sufficient to confer cardio-protection against pressure-overload induced cardiac dysfunction, but rather confers an unexpected long-term pro-survival effect in mice.

### Cardiac specific manipulation of BCAA catabolism led to transcriptome changes

**Fig. 7:**
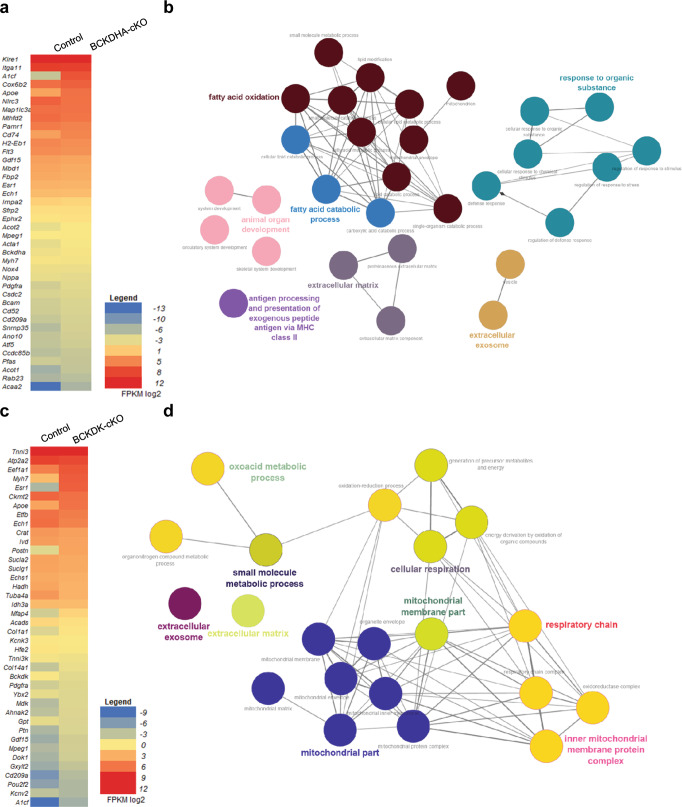
Transcriptome changes in BCKDHA-cKO and BCKDK-cKO mouse hearts. **a** Heatmap of selected genes detected in the Control vs the BCKDHA-cKO hearts from RNA-seq dataset ranked based on their expression levels in the BCKDHA-cKO hearts. **b** Integrated biological pathway and Gene-Ontology analysis for the top 500 differentially regulated genes ranked by significance in *P* values were determined by ClueGO in the Control and the BCKDHA-cKO hearts. **c** Heatmap of selected genes detected in the Control vs the BCKDK-cKO hearts from RNA-seq dataset ranked based on their expression levels in BCKDK-cKO hearts. **d** Integrated biological pathway and Gene-Ontology analysis for the top 500 differentially regulated genes ranked by significance in *P* values were determined by ClueGO in the Control and the BCKDK-cKO hearts. Colors represent related GO term families and only GO terms with *q* < 1E-20 are included.

Kruppel-like factor 15 (KLF15) is a key transcription factor regulating lipid, glucose and amino acids metabolism, including BCAA. Earlier studies have also identified branched chain amino acid could regulate KLF15 itself in a negative feedback loop [4, 31]. However, a transcription regulatory scheme specific to BCAA alternation has not been established. Consequently, the molecular mechanism in BCAA mediated regulation of cardiac contractile function remains to be established. To provide unbiased characterization of the impact of BCAA catabolic defect on cardiac transcriptome, we profiled transcriptome signatures in the hearts from the two mouse models by total RNA-sequencing. Differentially expressed genes between the Control and BCKDHA-cKO hearts were identified by QESEQ2 as described in Methods (Fig. 7a). Principal component analysis (PCA) of transcriptome landscape in the Control and BCKDHA-cKO hearts revealed a profound shift in cardiac gene expression upon E1α inactivation in both male and female mice (data not shown), with the female hearts showing more intra-group variations than the male counterparts. Among the top 500 significantly differentially expressed genes ranked based on *P* values, cellular pathway integrated GO enrichment analysis identified fatty acid utilization and oxidation, stress response and extracellular matrix remodeling as the most prominent cellular processes affected by BCAA catabolic defect in heart (Fig. 7b). In parallel, transcriptome profiling revealed that inactivation of BCKDK in heart led to a decrease in cardiac contractile and calcium regulating genes, including Tnni3, SERCA2 and Myh7 (Fig. 7c). Among the top 500 significantly differentially expressed genes ranked based on *P* values, cellular pathways GO enrichment has identified mitochondrial respiration genes to be the most significantly impacted upon BCKDK inactivation (Fig. 7d). These data offers potential link between BCAA catabolic activity and other cellular processes in heart.

## Discussion

To date, numerous studies have demonstrated the importance of BCAA catabolism in cardiac physiology, including suppressed BCAA catabolic genes in mouse and human failing hearts associated with elevated circulating BCAA levels [4]. To support the causal role of BCAA catabolism in cardiac pathological remodeling, both whole-body genetic suppression and systemic pharmacological activation of BCAA catabolism have supported the notion that BCAA catabolic activation has beneficial effect in heart failure [4, 12]. However, the key question remains whether lowering BCAA itself protects the cardiomyocyte under pathological stress and whether there is cell autonomous effect of BCAA catabolism in myocyte contractile regulation. In current study, we have generated two mouse lines to achieve cardiomyocytes specific BCAA activation or inactivation and demonstrated for the first time there is indeed a cardiomyocyte specific role of BCAA catabolism in cardiomyocyte dysfunction. Our results support the concept that suppression of BCAA catabolism in cardiomyocyte directly leads to cardiac dysfunction, but also raises interesting question why cardiomyocyte specific activation of BCAA catabolism fails to protect the heart from pressure overload induced heart failure. Our two mouse models will further serve as valuable tools to dissect the BCAA catabolic pathway and related metabolite function in cardiac physiology and pathological remodeling.

The pathogenic role of BCAA catabolic defect in heart failure as demonstrated in this study raises many new questions about the underlying mechanisms. Different hypotheses have been proposed, including accumulated BCAA, BCKA and loss of downstream metabolites for their bioenergetic contribution. The signaling effect of BCAA, BCKA and a downstream metabolite β-hydroxyisobutyrate (HiB) have been demonstrated in different cell types including mTOR regulation, ROS production, insulin sensitivity and mitochondrial function [20, 32]. Our results showed that BCAA/BCKA accumulation was prominent in heart tissue following BCAA challenge, consistent with a detrimental effect from elevated BCAA/BCKA level for cardiomyocyte function. Our result is also supported by a recent GWAS study where a meta analysis has identified a total of 47 risk loci associated with heart failure [33]. Among them, colocalization, gene expression profiling as well as Mendelian randomization provided convergent evidence for the roles of BCKDHA and circulating branched-chain amino acids in heart failure and cardiac structure, providing additional genetic evidence that BCKDHA plays a critical role in human heart failure development. The transcriptome reprogramming observed in the BCKDHA-cKO hearts highlights the potential mechanisms involved in the contribution of BCAA catabolic defects to heart failure. Bioenergetic disruption is a hallmark in failing hearts, combining with results from functional measurements, our findings in the BCKDHA-cKO hearts support a potential role of BCAA catabolic activity in the regulation of lipid metabolism and bioenergetics in heart. The underlying molecular mechanism linking BCAA/BCKA accumulation with pathological manifestation is still largely unknown. While KLF15 and mTOR signaling are both implicated in previous reports, the mouse model established in this study will allow further studies to validate and explore other unknown players. On the other hand, our finding on the systemic impact of cardiac BCAA catabolism is entirely unexpected. It implies that, at least under BCAA challenge, cardiac BCAA catabolic activity has a significant contribution to systemic BCAA homeostasis. This observation raises interesting question on the potential impact of cardiac BCAA defect on global metabolic health, and the potential contribution of extra-cardiac effect of BCAA on cardiac phenotype observed here, particularly in light of the recent report on the effect of extra-cardiac BCAA catabolism on blood pressure and heart failure [11].

Considering the potent effect of pharmacological inhibition of BCKDK on heart failure progression [4, 28, 34], our results with cardiac specific BCKDK inactivation model is quite puzzling, but consistent with the similar observation reported by Murashige et al. [11]. One possibility is that pharmacological approach by administration of small molecule BT2 systemically changes both cardiac as well as systemic BCAA flux, leading to changes of glucose homeostasis and whole body metabolism as previously demonstrated [35]. Therefore, the cardiac protective effect post injury could be a combined effect of both cardiac and systemic changes in BCAA flux. A second possibility is that the small molecule BT2 could target multiple downstream targets in addition to BCKDK. One approach to test this hypothesis may need to employ a global BCKDK depletion model, and to determine if the global BCKDK knockout could have any impact on cardiac function post pressure overload or myocardial infarction injury. The recent report about the modest effect of BCKDK inhibition on blood pressure regulation is both intriguing and potentially important. However, it is difficult to envision a modest peripheral blood pressure modulation can exert such profound impact on pressure overload induced cardiac dysfunction where the afterload is artificially induced through a surgical procedure proximal to the heart.

In short, this in vivo study using tissue specific manipulation of BCAA pathway has revealed a potent and cardiac specific role in the pathogenesis of heart failure, but also raises new questions about the mechanisms involved. The establishment of these two mouse models would provide an excellent in vivo system to investigate these questions in a temporal and cell-type specific manner.

## Supplementary information

Supplementary Figure 1

Supplementary Figure 2

Supplementary Figure 3

Supplementary Figure legend
