# Supplementary figures and images for: Cell-autonomous effect of cardiomyocyte branched-chain amino acid catabolism in heart failure in mice
Source: Acta Pharmacol Sin. 2023 Mar 29;44(7):1380–90. doi: 10.1038/s41401-023-01076-9 (PMC10310802; doi:10.1038/s41401-023-01076-9)

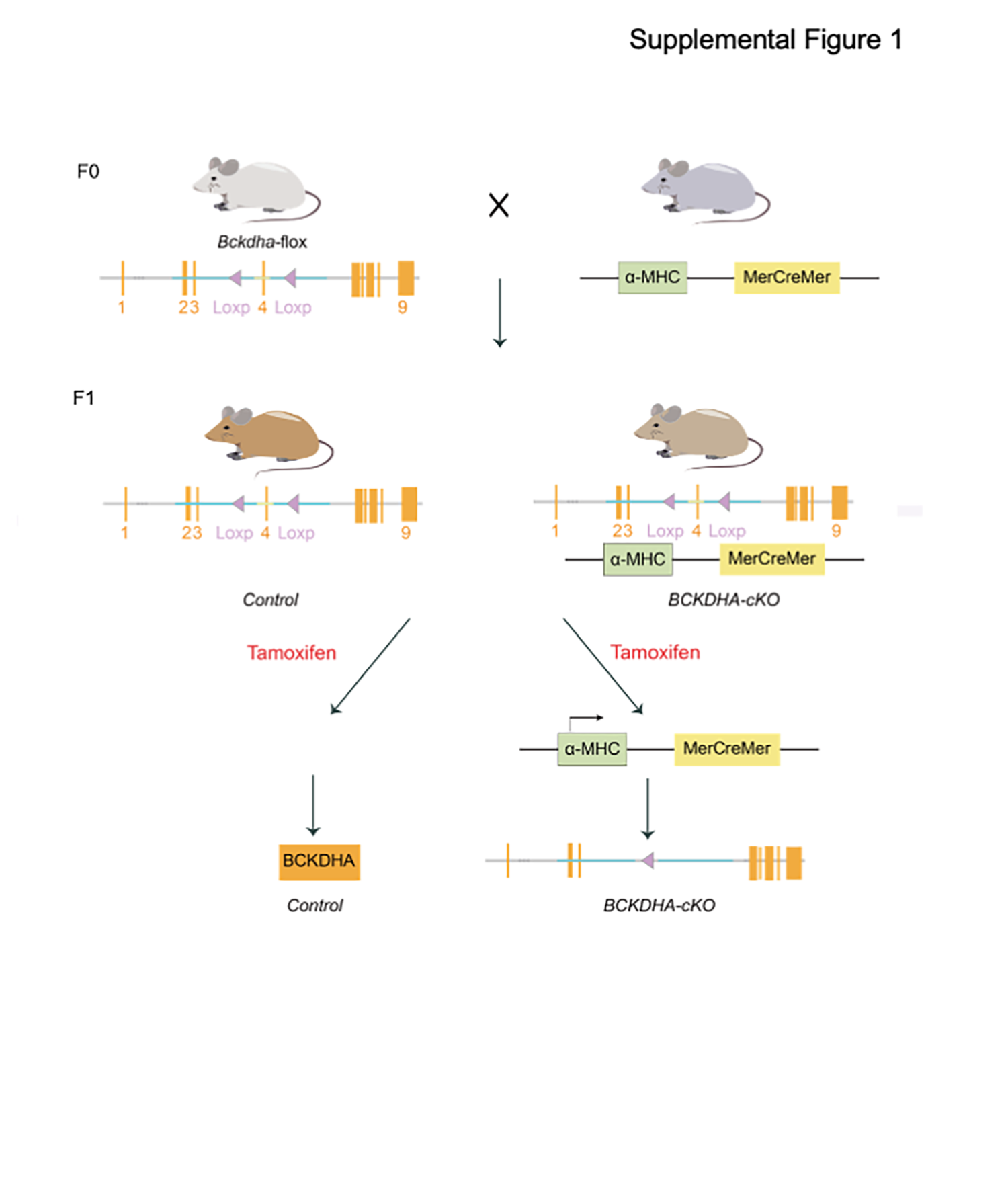

Supplement: Supplementary file 1 — Supplementary Figure 1 [file 41401_2023_1076_MOESM1_ESM.tif]

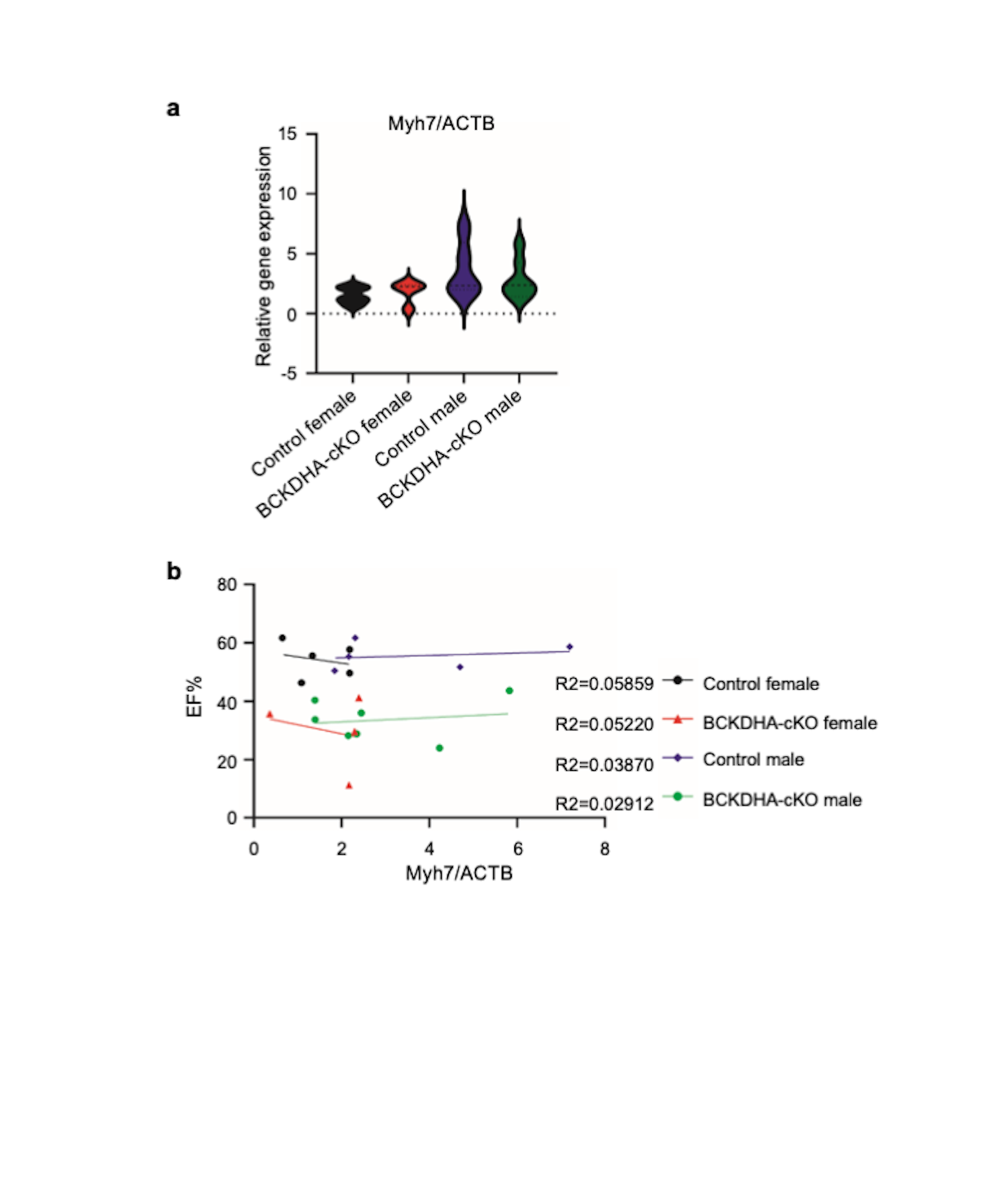

Supplement: Supplementary file 2 — Supplementary Figure 2 [file 41401_2023_1076_MOESM2_ESM.tif]

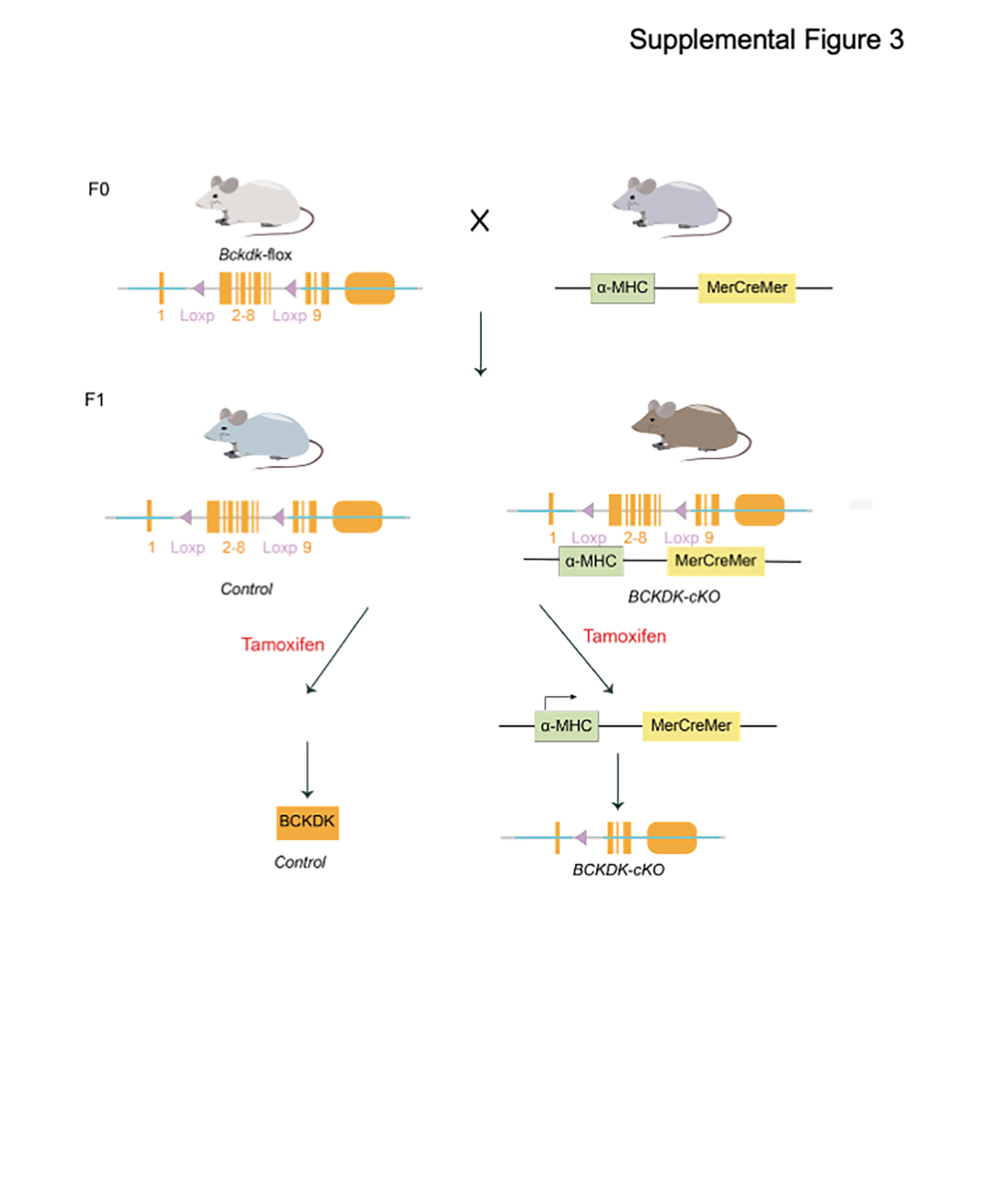

Supplement: Supplementary file 3 — Supplementary Figure 3 [file 41401_2023_1076_MOESM3_ESM.tif]
