# Supplementary material for: Cell-autonomous effect of cardiomyocyte branched-chain amino acid catabolism in heart failure in mice
Source: Acta Pharmacol Sin. 2023 Mar 29;44(7):1380–90. doi: 10.1038/s41401-023-01076-9 (PMC10310802; doi:10.1038/s41401-023-01076-9)
Supplement: Supplementary file 4 — Supplementary Figure legend [file 41401_2023_1076_MOESM4_ESM.docx]

**Supplemental Figure 1:** Illustration for the generation of *mbckdha* cardiac specific knockout mouse model.

**Supplemental Figure 2: Myh7 expression in BCKDHA-cKO male and female mouse hearts a.** mRNA levels of Myh7 relative to ACTB in the Control and the BCKDHA-cKO male and female mouse hearts. **b** Quantitative correlation of Myh7 mRNA expression level with cardiac function in each cohort, n=4-7 per group

**Supplemental Figure 3:** Illustration for the generation of m*bckdk* cardiac specific knockout mouse model.
